# Temporomandibular Joint Dysfunctions: A Systematic Review of Treatment Approaches

**DOI:** 10.3390/jcm12124156

**Published:** 2023-06-20

**Authors:** Blanca González-Sánchez, Pablo García Monterey, María del Valle Ramírez-Durán, Elisa Mª Garrido-Ardila, Juan Rodríguez-Mansilla, María Jiménez-Palomares

**Affiliations:** 1 ADOLOR Research Group, Department of Medical-Surgical Therapy, Faculty of Medicine and Health Sciences, Extremadura University, 06006 Badajoz, Spain; blgonzalezs@unex.es (B.G.-S.); jrodman@unex.es (J.R.-M.); mariajp@unex.es (M.J.-P.); 2 Department of Medical-Surgical Therapy, Faculty of Medicine and Health Sciences, Extremadura University, 06006 Badajoz, Spain; pablogarciamonterrey.fisio@gmail.com; 3 Department of Nursing, Centro Universitario de Plasencia, Universidad de Extremadura, 10600 Plasencia, Spain

**Keywords:** temporomandibular joint, physical therapy modalities, temporomandibular joint disorders, therapeutics

## Abstract

Temporomandibular disorders (TMDs) encompass a diverse array of conditions affecting both the structure and function of the jaw. The aetiology of TMDs is multifactorial and may arise from muscular and joint disorders, degenerative processes, or a combination of various symptoms. The objective of this review was to analyse the physiotherapy treatment techniques used for the management of temporomandibular disorders. This review also aimed to compare the effectiveness of the differenttreatment methods used and identify the dysfunctions for which physiotherapy interventions are applied as the main treatment. A systematic literature review was conducted using the PubMed, ScienceDirect, Dialnet, and PEDro databases. After applying the inclusion criteria, 15 out of 656 articles were included. The application of different physiotherapy techniques, both alone and in combination, is effective in controlling the primary symptoms of TMD in patients. These symptoms include pain, functionality, and quality of life. The use of physiotherapy as a conservative intervention method for TMDs is supported by sufficient scientific evidence. The combination of different therapies within physiotherapy achieves the best results in treatment. Therapeutic exercise protocols, in combination with manual therapy techniques, are the most commonly utilized method for addressing TMDs and thus provide the best results according to the analysed studies.

## 1. Introduction

The temporomandibular joint (TMJ) is a bilateral synovial joint located on both sides of the craniomandibular complex, which is involved in chewing, swallowing, speech, and other automatic movements such as yawning, grinding, or clenching; therefore, resting the joint is challenging under normal physiological conditions, except for clamping. Between 2000 and 2500 jaw movements occur every day approximately. Consequently, TMJ pathophysiology significantly impacts individuals’ ability to perform basic daily life activities and vital functions [1,2]. A broad range of disorders arise related to functional alterations of the TMJ structure, which have multifactorial causes such as biological, behavioural, emotional, cognitive, environmental, and social factors [3]. The most frequent pathology is “TMJ and masticatory muscle dysfunction” defined as any abnormal anatomical connection between the structures that create the joint. In this sense, when moving the joint, the anterior displacement of the articular disc is the most frequent cause of the clinical sign of the “click” during the collision between the articular disc and the condyle. [4,5]. Symptoms may include pain referred to the neck and head, ear involvement, dental wear, alteration of joint mobility, hypertrophy of masticatory musculature, as well as other signs such as inflammatory processes or noises when making mandibular movements [6]. The diagnosis of any TMJ dysfunction is derived from the evaluation of signs and symptoms. In addition, the most used and cited diagnostic classification systems are the Diagnostic Criteria for Temporomandibular Disorders (DC/TMD) and the American Academy of Orofacial Pain (AAOP) classification [7].

Regarding the DC/TMD, it consists of two axes: Axis I, which assesses the biological state of the structures, their dynamics, presence of pain, headache, joint noise, and closed and/or open locking of the jaw, and Axis II, which assesses the psychosocial state. [8]. Therefore, the comprehensive assessment of the TMD patient based on the biopsychosocial health model is the most appropriate for both the treatment and research of this condition. [7]. Furthermore, it should be carried out by a specialized multidisciplinary team, including speech therapy, dentistry, pharmacology, physiotherapy, psychology, and surgery when necessary [9,10]. Physiotherapists’ interventions should focus on relieving pain, improving strength and neuromuscular coordination, and increasing the joint’s range of motion. Physiotherapy interventions include manual therapy, exercise-based programs, electrotherapy techniques, or mobilizations, among others [10,11].

The objective of this review was to analyse which physiotherapy treatment techniques are used for the management of the temporomandibular disorders and compare their effectiveness as well as identify the dysfunctions for which physiotherapy interventions are applied as the main treatment.

## 2. Materials and Methods

### 2.1. Study Design

This systematic review was carried out following the PRISMA recommendations [12] and was registered in PROSPERO (International prospective register of systematic review) with register number CRD42022362936.

### 2.2. Search Strategy

For the realization of this work, a literature review was executed during the months of June and July 2022 in the following databases: PubMed, ScienceDirect, Dialnet, and PEDro.

The used MeSH terms were as follows: Temporomandibular joint, Physiotherapy, Temporomandibular joint disorders, Temporomandibular dysfunction syndrome, Treatment. The search strategies were as follows: “Temporomandibular joint AND physiotherapy”, “Temporomandibular joint disorders AND physiotherapy” and “Temporomandibular dysfunction syndrome AND physiotherapy”, translated into Spanish in those databases that required it. The strategies used in each database are shown in Table 1.

### 2.3. Eligibility Criteria

Criteria selection was established following the PICO model (population, intervention, control, and outcome).

Inclusion criteria were as follows:

- Studies published in Spanish or English.
- Studies published between 2010 and the present (June 2022).
- Clinical trial studies.
- Studies conducted in patients diagnosed with temporomandibular disorders who have undergone treatment.

Exclusion criteria were as follows:

- Duplicated studies.
- Studies in which no type of treatment was studied.

### 2.4. Study Selection

After conducting a search for studies and applying filters, the titles and abstracts of the identified studies were reviewed to determine their eligibility for inclusion in this review. Articles that met the inclusion criteria underwent a detailed examination following an initial screening.

### 2.5. Methodological Quality Analysis

To assess the methodological quality of clinical trials included in this bibliographic review, the “PEDro” scale was used [13]. This scale was developed by Verhagen in 1998 and is based on the Delphi list. The scale comprises 11 items, with the first item pertaining to external validity being excluded from calculating the total score. Points are awarded only when the clinical trial meets the criteria. The maximum achievable score on this scale is 10 points, indicating excellent methodological quality. Studies with a score of less than 4 points are classified as having low methodological quality, while those with a score between 4 and 5 points are of medium quality. Clinical trials with a score between 6 and 8 points are considered to have good methodological quality.

## 3. Results

**Figure 1:**
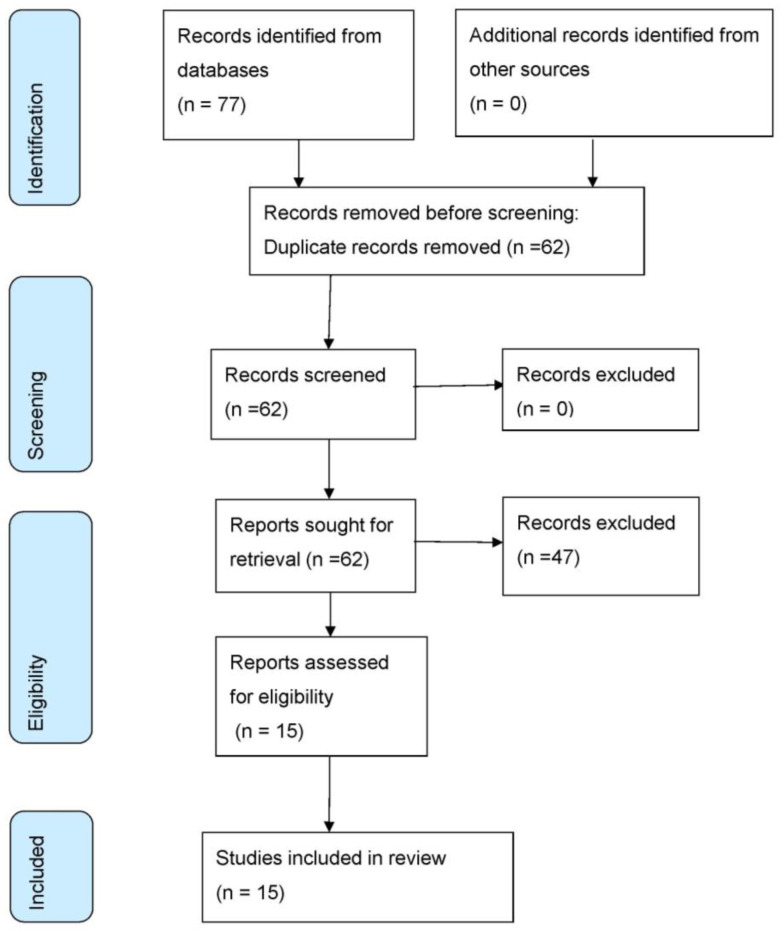
PRISMA flow diagram.

A total of 103 studies were found from the search of all databases; duplicate records were excluded, and 65 studies were selected. The final sample consisted of 15 studies. The study selection process is shown in the PRISMA flow diagram (Figure 1).

Table 2 shows the characteristics of the studies included in this review regarding objectives, sample, intervention, duration, assessment tools utilized, and results of each of the clinical trials.

The clinical trials selected for this study aim to provide a broad range of therapeutic possibilities for patients suffering from TMDs. Brochado et al. [14] evaluated the effectiveness of manual therapy and photobiomodulation, both independently and in combination. Delgado de la Serna et al. [15] investigated the effects of cervico-mandibular manual therapies, accompanied by therapeutic exercise and a health education program. Several articles have explored the effects of therapeutic exercise, such as by Abboud et al. [16] and Calixtre et al. [17], which evaluated the effects of a program based on cervical mobilization and therapeutic exercise. Craane et al. [18] investigated the effects of physiotherapy on pain and mandibular dysfunction associated with disc displacement. Finally, Madani and Mirmotazavi [19] compared three treatment methods in patients with painful TMJ clicks.

Considering the DC/TMD [8], and despite the absence of reporting the cause of the TMD in several studies [17,21,22,23,24,25], we found TMD of myogenic origin in Brochado et al. [14] and in the one developed by Urbanski et al. [27], in which participants present TMDs with a muscular component. Joint pain as a disorder was found in two of the included studies [19,20]. In the research conducted by Delgado de la Serna et al. [15], the researchers analyzed the relationship between Tinnitus and TMD. There are two studies that observed that patients with TMD might have a joint cause, either due to ankylosis [28] or after arthroscopy [16]. Only the work of Craane B et al. [18] includes patients with TMD with anterior displacement of the disc, and it is the work of Brandao et al. [26] the only one in which we find patients with TMD associated with a limitation or not of the oral opening. Several authors have assessed the effectiveness of various interventions for patients with myofascial pain. Dalewski et al. [20] examined three intervention modalities: nonsteroidal anti-inflammatory drugs, dry needling, and a control group. However, Dib-Zakkour et al. [21] investigated the effectiveness of dry needling as an initial treatment step for TMDs. Another emerging treatment option was the use of electroacupuncture for TMJ pain. Nambi et al. [20] conducted a clinical trial to investigate the efficacy of this technique by dividing the sample into subjects who received this treatment and those who received a placebo effect combined with physiotherapy.

In the area of electrotherapy, a number of studies have been conducted to investigate the effects of various therapeutic techniques on TMDs. Chellappa and Thirupathy [23] carried out a trial in which TENS and low-level laser therapy were administered to subjects with this condition. Li et al. [24] investigated the effect of two other techniques, extracorporeal shock waves (ESW), and shortwave (UW). Jo et al. [25] aimed to evaluate the efficacy of PRF therapy by randomly assigning subjects to receive either pulsed radiofrequency or a placebo effect, in conjunction with other conventional treatments. Brandao and Mendes [26] conducted a study to investigate the effects of an isotonic exercise-based treatment program for pain relief in patients with temporomandibular disorders. Similarly, Urbanski et al. [27] conducted a study to compare the degree of relaxation achieved in the anterior part of the temporal muscles and masseter muscles through the use of post-isometric relaxation and myofascial release techniques.

Several researchers have examined the impact of administering intramuscular injections of botulinum toxin to the masticatory muscles of individuals suffering from temporomandibular joint ankylosis. For instance, Shandilya et al. [28] conducted a study in which participants were allocated to two groups: the first group received botulinum toxin injections prior to surgery, while the second group, the control, were given injections of saline solution.

### Methodological Quality Assessment

The methodological quality of the studies included in this review was analysed using the PEDro scale [13]. Based on this, out of the fifteen articles included in this review, nine articles [14,15,16,17,18,19,20,25,27] exhibit good methodological quality. Three of the fifteen articles [21,23,24] show regular quality, while two [26,27] exhibit poor quality, having obtained a score of 3 points. Lastly, the work carried out by Nambi et al. [22] presents the highest score among the 15 articles, 9/10, indicating excellent methodological quality. All 11 criterion items were met; however, there was no item that was completely checked by every study. The initial criterion, pertaining to the description of participant selection methods within the articles, is met by all the studies, with the exception of Brochado et al. [14]. However, only two of the fifteen articles [22,25] met the fifth criterion concerning subject blinding (see Table 3).

## 4. Discussion

This systematic review aimed to provide a comprehensive overview of the physiotherapy treatment techniques available for the management of the temporomandibular disorders, comparing the effectiveness of different treatment methods and identifying the dysfunctions that are most prone to physiotherapy interventions.

There are various physiotherapy modalities that could be considered as the first option in the treatment of TMDs. Manual therapy interventions have been found to produce positive results in relieving myofascial pain, pain in the masticatory muscles, and improving joint function [14,15,27]. These findings are consistent with those of Senbursa et al. [29], which demonstrated the efficacy of manual therapy in treating patients with supraspinatus muscle tendinopathies.

Similarly, therapeutic exercise protocols have been found to be beneficial in reducing pain and improving joint mobility [16,17,18,20,27]. Nejati et al. [30] also demonstrated the effectiveness of a physiotherapy program that includes therapeutic exercise in the treatment of sacroiliac dysfunctions, further supporting the use of therapeutic exercise as a technique for improving mobility and reducing pain. Is this line, it interesting to highlight that current research has shown that TMDs may be related to the pelvis in disorders such as endometriosis [31]. Furthermore, a recent study has revealed correlations between the presence of pain on the right and left sides of the pelvis and pain on the right and left sides of the TMJ, teeth clenching and TMJ pain, the occurrence of pelvic pain and the treatment modality for endometriosis performed, and the presence of pain in other parts of the body but the pelvis and the treatment that the patient received for endometriosis [31].

Delgado de la Serna et al. [15] demonstrated that the integration of both interventions into a treatment program leads to an improvement in symptomatology for patients with TMJ-attributed tinnitus. This finding is consistent with the work of Rodríguez-Sanz et al. [32], who also found positive results in the treatment of patients with chronic cervical pain.

The study by Dib-Zakkour et al. [21] demonstrated that the bilateral application of deep dry needling on the masseter muscle resulted in significant reductions in facial pain and muscle activity following trigger point needling. This finding is consistent with the results obtained by Ziaeifar et al. [33], whose study showed significant changes in neck pain intensity and disability levels after dry needling sessions. Furthermore, Nambi et al. [22] found that the combination of electrotherapy and dry needling is effective in reducing pain in patients with TMJ myalgias. Similar efficacy has been reported by other studies, such as the work by Lo et al. [34], which found that electroacupuncture significantly reduces pain and improves range of motion in patients diagnosed with frozen shoulder.

Lastly, the inclusion of anterior positioning splints in a treatment program can help reduce pain and sounds in patients with TMDs. The occlusal splint, which causes vertical condylar distraction and eliminates the occlusal factor that may be responsible for TMDs, is one of the most commonly used treatments [35]. However, Van Grootel et al. [36] found no significant differences between the use of splints and physiotherapy as initial treatment for such disorders.

Notwithstanding, although the different physical therapy treatments analysed in this review showed several benefits, it should be noted that, due to the heterogeneity of the signs and symptoms associated with TMDs, it is difficult to determine which is the most beneficial for TMDs depending on their origin, despite the fact that all the treatment modalities analysed seek to improve symptomatology and functionality.

### 4.1. Clinical Implications

Temporomandibular disorders and their associated pain are commonly encountered in clinical practice. Given their recurrent nature, they can have a negative impact on the patient’s social life [37]. Analysed studies suggest that identifying predictors of treatment outcome in patients (e.g., age, psychosocial impairment) may be more important than focusing solely on the disease [38]. The Diagnostic Criteria for TMJ Disorders (DC/TMD) have been used as a foundation for the development of a protocol for evaluating patients with TMDs for both clinical and research purposes [39].

### 4.2. Limitations

Numerous techniques are used in the treatment of TMDs, making it challenging to determine the most effective approach. Restricting the inclusion criteria to obtain the most current sample may have resulted in a reduction in sample size, as well as a decrease in the variety and quantity of treatments examined. However, the inclusion criteria were established to include studies conducted in patients diagnosed with temporomandibular disorders who have undergone treatment, and therefore including the different classifications of the Taxonomic Classification for Temporomandibular Disorders [8].

## 5. Conclusions

Upon analysing the studies included in this review, we have come to the conclusion that the primary treatment alternatives for physiotherapeutic management of various TMDs include manual therapy, therapeutic exercise-based programs, and different electrotherapy techniques. The use of physiotherapy as a conservative intervention method for the treatment of TMDs, such as temporomandibular pain, painful clicking, disorders of the masticatory musculature, articular disc disorders, and psychosocial issues, is well-supported by sufficient scientific evidence.

The scientific evidence further indicates that a combination of different therapeutic approaches within physiotherapy leads to the best results in the treatment of TMDs. However, according to the analysed studies, the most commonly used method for addressing TMDs is the use of therapeutic exercise protocols in combination with manual therapy techniques, which yields the best results.

## Figures and Tables

**Table 1:** Syntax of combined descriptors in the scientific database search.

Data Bases	Searching Strategies
PubMed	Temporomandibular joint AND physiotherapy Temporomandibular joint disorders AND physiotherapy Temporomandibular dysfunction syndrome AND physiotherapy
ScienceDirect	Temporomandibular joint AND physiotherapy Temporomandibular joint disorders AND physiotherapy Temporomandibular dysfunction syndrome AND physiotherapy
Dialnet	Articulation temporomandibular AND physiotherapy Trastornos de la articulación temporomandibular AND fisioterapia Síndrome de disfunción temporomandibular AND fisioterapia
PEDro	Temporomandibular joint AND physiotherapy Temporomandibular joint disorders AND physiotherapy Temporomandibular dysfunction syndrome AND physiotherapy

**Table 2:** Characteristics of the studies included in this review.

Study	Objective	Diagnosed Disorder [8]	Sample/Age	Intervention	Duration	Assessment Tool	Results
Brochado et al., 2018 [14]	To investigate the efficacy of manual therapy and photobiomodulation, separately or in combination, with regard to pain intensity, mandibular movements, psychosocial aspects, and anxiety in patients with TMDs.	TMD (Myogenic and arthrogenic)	51 patients 21 years old or more.	G1: 18 patients. Photobiomodulation application. G2: 16 patients. Manual therapy G3: 17 patients. Both techniques.	12 sessions. Duration unspecified.	Visual Analog Scale (VAS). Beck Anxiety Inventory (BAI). Research Diagnostic Criteria for TMJ Disorders (RDC/TMD).	All protocols demonstrated changes in the reduction of pain (*p* < 0.001) and anxiety symptoms (*p* ≤ 0.05). The combination of both methods does not prove to be more effective than both separately.
Delgado de la Serna et al., 2020 [15]	To investigate the effects of adding cervical-mandibular manual therapies to an educational and exercise program in individuals with tinnitus associated with TMDs.	TMD with associated tinnitus	61 patients. 18–65 years.	Cranio-cervical treatment, TMJ treatment, self-massages exercises and patient education.	Six 30 min sessions during a month.	Intensity of TMJ pain. Tinnitus disability inventory. Disability related to TMJ disorders. Beck depression inventory (BDI-II) Mandibular joint range.	Cervico-mandibular manual therapies in combination with exercise/education produced better results than the application of exercise/education alone in people with TMJ-attributed tinnitus (*p* < 0.001).
Abboud et al., 2018 [16]	To compare two physiotherapy programs, progressive versus immediate, after TMJ arthroscopy.	TMD (TMJ arthroscopy)	137 patients Mean age 27.5 gradual group mean age 28.5 immediate group	G1: 68 patients gradual exercise program. G2: 69 patients immediate exercise program.	10 months post-op rehabilitation. Sessions unspecified.	VAS Maximum Mandibular Opening Range (MMO)	Group 2 showed better results, in terms of pain and MMO, compared to group 1.
Calixtre et al., 2016 [17]	To investigate the effects of a program based on cervical mobilization and therapeutic exercise in patients with TMDs.	TMD	12 patients. Mean age = 22.08 (SD ± 2.23)	Subjects were evaluated 3 times: twice pre-intervention and once post-intervention 3–5 days after the last session.	10 physiotherapy sessions for 5 weeks. Duration unspecified	Cohen coefficient. Mandibular Functional Impairment Questionnaire. MMO Pressure pain threshold in both masseter and temporalis.	The protocol demonstrates significant changes in pain after MMO (32.3–8.8 mm to 38–8.8 mm), referred pain and joint functionality in such patients.
Craane et al., 2012 [18]	To evaluate the effects of physiotherapy on pain and mandibular dysfunction associated with anterior disc displacement without reduction of the joint space.	TMD with Anterior disc displacement without reduction of the temporomandibular joint (closed lock)	49 patients Mean age 34.7 control group, mean age 38.5 experimental group.	G1: 23 patients. Therapeutic exercise. G2: 26 patients control group.	1 year. Number and duration of sessions unspecified.	No use of quantitative scales	Physical therapy had no significant additional effect in patients with anterior disc displacement, without reduction, of the TMJ.
Madani et al., 2011 [19]	To evaluate the efficacy of three treatment methods in patients with painful TMJ clicking.	TMD (joint pain)	60 patients Mean Age 27.2 G1 23.15 G2 22.43 G3	G1: anterior positioning splint. G2: physical therapy. G3: physical treatment and splinting. Group ratio 1:1:1.	4 weeks 35 min per session.	RDC/TMD VAS Bilateral palpation of TMJ muscles.	Subjective pain decreased in all three groups (*p* < 0.05). Changes between group 1 and 2 (*p* < 0.05). Group 2 and 3 no change. The anterior positioning splint proved to be the best treatment to reduce mandibular pain and sounds in patients with TMJ problems.
Dalewski et al., 2019 [20]	To compare the early effectiveness of routine intervention methods in patients with chronic orofacial pain.	TMD (joint pain)	90 patients. 18–65 years.	G1: Occlusal appliance (OA) + NSAIDs. G2: OA + dry needling (DN). G3: OA (control group).	3 weeks. Number and duration of sessions unspecified.	VAS Sleep Activity and Pain Questionnaire (SPAQ).	OA + NSAIDs showed better relief of orofacial pain, compared with the use of OA alone or together with DN. No differences between pain perception and quality of life between the OA + DN and OA groups.
Dib-Zakkour et al., 2022 [21]	To determine the effectiveness of deep dry needling as the first step in the treatment of TMDs.	TMD	36 patients. 18–40 years.	Experimental group: bilateral dry needling of the masseter muscle. Control group: simulation of the technique.	15 days. Evaluated pre-puncture, 10 min post puncture and after 15 days.	VAS Bilateral muscle palpation. T-Scans. Electromyography.	Facial pain is significantly reduced and is accompanied by a marked reduction in muscle activity after the puncture of its trigger points.
Nambi et al., 2022 [22]	To investigate the clinical and functional efficacy of electroacupuncture therapy on TMJ pain with orofacial myalgia after healing in patients with cervicofacial burns.	TMD with a cervico-facial burn	30 patients. Age unspecified	Active group: 15 patients underwent electroacupuncture. Placebo group: 15 patients	4 sessions/week for 1 month.	Pain intensity/threshold/frequency and mouth opening, disability level, and quality of life were measured at baseline, after week 4, week 8, and 6-month follow-up	Electroacupuncture therapy has an ideal treatment protocol for TMJ pain with orofacial myalgia after a healed cervicofacial burn (*p* < 0.001).
Chellappa et al., 2020 [23]	To compare the therapeutic efficacy of transcutaneous electrical nerve stimulation (TENS) and low-level laser in patients with TMDs	TMD	60 patients Age unspecified.	All patients underwent both treatment methods.	6 weeks. Low level laser: 2 sessions/3 weeks. TENS: 2 sessions/3 weeks, 15 min/session	VAS Numerical Rating Scale (NRS) Palpation of the bilateral masticatory musculature	The results of low-level laser therapy were significantly superior to those obtained with TENS (*p* < 0.01).
Li et al., 2020 [24]	To compare the therapeutic efficacy of extracorporeal shock waves (ESW) and ultrashort waves (UW) for TMDs.	TMD	80 patients. >18 years.	G1: ESW. G2: UW	1 month. Sessions unspecified	VAS MMO	ESW presents better results than UW (*p* < 0.05).
Jo et al., 2021 [25]	To evaluate the long-term efficacy and patient satisfaction with pulsed-radiofrequency therapy (PRF) in TMDs.	TMD	86 + 23 women 18–65 years old	G1: PRF G2: placebo.	12 weeks. 1 session/week 10min duration.	VAS MMO Baseline, after 4, 8, and 12 weeks.	PRF significantly reduced TMD pain. The effect was long-lasting after completion of treatment relative to the placebo group.
Brandão et al., 2021 [26]	To investigate the electroneurophysiological aspects of volunteers with TMDs before and after performing isotonic exercises for pain relief.	TMD with or without opening limitations	23 patients 18–60 years old	Experimental: 12 patients Control: 11 patients.	4 weeks 2 session/week	Electroencephalography RDC/TMD	Isotonic exercises performed to reduce pain provided a small increase in alpha power density in the left temporal, parietal, and occipital regions.
Urbański et al., 2021 [27]	To compare the degree of relaxation of the anterior part of the temporal muscles and the masseter muscles achieved through the use of post-isometric relaxation and myofascial release methods in patients requiring prosthetic treatment due to TMDs with a dominant muscular component.	TMD with a dominant muscular component	60 patients 19–40 years old.	G1: post-isometric relaxation treatment. G2: myofascial release treatment.	2 weekly sessions. 30 min duration.	EMG masticatory musculature. VAS	No significant changes were found between the two groups.
Shandilya et al., 2020 [28]	To determine the efficacy of intramuscularly infiltrating botulinum toxin into the masticatory muscles in patients with TMJ ankylosis.	TMD (TMJ ankylosis)	20 patients. 18–45 years old.	Intervention group: botulinum toxin infiltration prior to surgery. Control group: saline solution.	6 months. Evaluated after 1 week, 1, 3, and 6 months.	EMG.	Botulinum toxin infiltrations showed better results in terms of pain during mandibular opening exercises compared to the control group.

**Table 3:** Evaluation of the methodological quality of the studies.

Study	1	2	3	4	5	6	7	8	9	10	11	Points
Brochado et al., 2018 [14]	N	Y	N	Y	N	N	Y	N	Y	Y	Y	6/10
Delgado de la Serna et al., 2020 [15]	Y	Y	Y	Y	N	N	Y	Y	Y	Y	Y	8/10
Abboud et al., 2018 [16]	Y	Y	N	Y	N	N	N	Y	Y	Y	Y	6/10
Calixtre et al., 2016 [17]	Y	N	N	Y	N	S	Y	N	Y	Y	Y	7/10
Craane et al., 2012 [18]	Y	Y	Y	Y	N	N	Y	Y	Y	Y	Y	8/10
Madani et al., 2011 [19]	Y	Y	N	Y	N	N	N	Y	N	Y	Y	6/10
Dalewski et al., 2019 [20]	Y	Y	Y	Y	N	N	Y	Y	N	Y	Y	7/10
Dib-Zakkour et al., 2022 [21]	Y	N	N	N	N	N	N	Y	Y	Y	Y	5/10
Nambi et al., 2022 [22]	Y	Y	Y	Y	Y	N	Y	Y	Y	Y	Y	9/10
Chellappa et al., 2020 [23]	Y	Y	N	Y	N	N	N	N	N	Y	N	4/10
Li et al., 2020 [24]	Y	Y	N	N	N	N	N	N	Y	Y	Y	5/10
Jo et al., 2021 [25]	Y	Y	Y	Y	Y	Y	N	N	Y	Y	Y	8/10
Brandão et al., 2021 [26]	Y	Y	N	N	N	N	N	N	N	Y	N	3/10
Urbański et al., 2021 [27]	Y	N	N	Y	N	N	N	N	N	N	Y	3/10
Shandilya et al., 2020 [28]	Y	N	N	Y	N	N	N	Y	Y	Y	Y	6/10

## Data Availability

Data is available upon reasonable request to the authors.
